# Chronic lung disease of prematurity and bronchopulmonary dysplasia

**DOI:** 10.36416/1806-3756/e20240279

**Published:** 2024-09-16

**Authors:** Gabriela de Azevedo Bastian de Souza, Maria Paula Hanel, Eduardo da Costa Herter, Leonardo Araujo Pinto, Marcus Herbert Jones

**Affiliations:** 1. Centro Infant, Escola de Medicina, Pontifícia Universidade Católica do Rio Grande do Sul, Porto Alegre (RS) Brasil.; 2. Programa de Pós-Graduação em Pediatria e Saúde da Criança, Pontifícia Universidade Católica do Rio Grande do Sul - PUCRS - Porto Alegre (RS) Brasil.

## DEFINITION AND PHENOTYPES

Chronic lung disease of prematurity (CLDP) refers to various respiratory disorders resulting from prematurity. With the continuous rise in birth and survival rates of premature infants, the incidence of CLDP has increased. In Brazil, 11.94% (302,528) of live births in 2023 were premature (born at < 37 weeks of gestation), and 0.59% (15,134) were extremely premature (born at < 28 weeks).^1^ This improved survival of extremely preterm newborns is largely due to advancements in neonatal intensive care over recent decades. Strategies to enhance preterm infant survival include new antimicrobials, corticosteroid therapy for high-risk pregnancies, artificial surfactants, and assisted ventilation methods that are more effective, like CPAP.^2^

Although survival rates have improved, morbidity and mortality have shifted to later stages, often due to sequelae of prematurity such as CLDP, which involves changes in lung structure, including altered septation, vascularization, and a reduced number of alveoli. Those changes, which lead to impaired gas exchange and reduced lung function, can persist into adulthood.^2^ ^,^ ^3^

As detailed in Chart 1, CLDP encompasses a spectrum of respiratory conditions in premature infants, from minimal symptoms to severe conditions like bronchopulmonary dysplasia (BPD). Inflammatory processes significantly influence those conditions, with cytokine storms exacerbating disease progression. Medical interventions, particularly invasive ventilation, contribute to pro-inflammatory cascades and play a crucial role in CLDP pathogenesis.

**Chart 1:** Differences in definition and management between chronic lung disease of prematurity and bronchopulmonary dysplasia.

Disease	Definition	Management
CLDP	This comprises a spectrum of respiratory pathologies resulting from prematurity, ranging from minimal respiratory symptoms with some loss of lung function to more severe cases. Viral bronchiolitis caused by RSV or other viral infections, recurrent wheezing, and asthma in school-age children are major causes of morbidity associated with CLDP.	• RSV prevention
		• Inhaled steroids for patients with atopy and symptoms of asthma
		• The use of immunostimulants like bacterial lysate as an option to prevent recurrent respiratory infections
BDP	This is the most severe clinical complication within the CLDP spectrum, defined as the need for supplemental oxygen or ventilation for 28 days or more from birth to 36 weeks post-conception.	• Follow-up with pulmonary function tests
		• RSV monoclonal antibodies
		• Asthma treatments only for specific groups
		• If supplemental oxygen use is recommended, a target saturation of >90%

CLDP: chronic lung disease of prematurity; RSV: respiratory syncytial virus; and BDP: bronchopulmonary dysplasia.

During the intrauterine period, airway development progresses through several stages. Many preterm infants are born during the saccular stage (24-36 weeks gestation), with compromised surfactant production and underdeveloped bronchioles and airways. Prenatal factors, such as fetal growth and gestation duration, and postnatal factors, such as ventilatory interventions, can significantly affect lung development into adulthood.^2^

The most severe form of CLDP is BPD, defined by the need for supplemental oxygen or ventilation for 28 days or more, up to 36 weeks post-conception. The etiology of BPD is multifactorial, with inflammation and prolonged mechanical ventilation as key contributors. It is characterized by chronic respiratory disease, with consequences lasting beyond the neonatal period. Children with BPD are at higher risk for lower respiratory tract infections, airway hyperresponsiveness, and hospitalization in the first two years of life.

Within BPD, there are various phenotypes. Some preterm infants exhibit significant lung function impairment without meeting the criteria for BPD or showing signs of neonatal respiratory disease. Others may have severe lung disease, requiring long-term mechanical ventilation or oxygen supplementation. With less aggressive neonatal ventilation techniques, the histological phenotype of BPD has shifted from post-traumatic conditions to a form characterized by arrested alveolar development. Recent studies suggest that patients with BPD are at increased risk for subclinical pulmonary hypertension, exercise-induced lung disease, hypertension, right ventricular dysfunction, and autonomic dysfunction.

Premature birth alters lung structure, increasing vulnerability to acute viral infections in early life. These infections, typically mild in full-term infants, are more severe in preterm infants, with a higher risk of hospitalization and ventilatory support, particularly in infants infected with respiratory syncytial virus (RSV). Many preterm infants develop recurrent wheezing and asthma-like symptoms, often treated with bronchodilators and inhaled steroids, though the response is generally poor. Symptom management should be tailored to individual severity, including RSV prevention and inhaled steroids for patients with atopy and asthma symptoms. Immunostimulants like bacterial lysates may also be considered.

## MANAGEMENT

Various treatments for BPD are under study, including inhaled or systemic corticosteroids, bronchodilators, and supplemental oxygen, though interventional studies are limited.^4^ The 2020 European Respiratory Society consensus recommends lung function tests for monitoring BPD in children, with pulmonary imaging reserved for severe cases. Bronchodilators should be used for patients with recurrent hospitalizations, exercise intolerance, or asthma-like symptoms, while inhaled corticosteroids should not be used for BPD treatment. If supplemental oxygen is needed, the target saturation should be at least 90%.^4^ Palivizumab and, in the future, nirsevimab, can be used to prevent severe RSV infection in high-risk populations, including those with congenital heart disease, BPD, and prematurity.^5^

The main limitations in BPD treatment stem from the scarcity of high-quality studies, leading to a low level of evidence for many guidelines. More prospective studies are needed in order to monitor development of preterm children into school age and adulthood, as well as randomized clinical trials to determine the most effective treatments.^4^
